# Health related quality of life among children with transfusion dependent β-thalassaemia major and haemoglobin E β-thalassaemia in Sri Lanka: a case control study

**DOI:** 10.1186/s12955-019-1207-9

**Published:** 2019-08-08

**Authors:** Sachith Mettananda, Hashan Pathiraja, Ravindu Peiris, Dayananda Bandara, Udaya de Silva, Chamila Mettananda, Anuja Premawardhena

**Affiliations:** 1 Department of Paediatrics, Faculty of Medicine, University of Kelaniya, Thalagolla Road, Ragama, 11010 Sri Lanka; 2 Colombo North Teaching Hospital, Ragama, Sri Lanka; 3 Kurunegala Teaching Hospital, Kurunegala, Sri Lanka; 4 Anuradhapura Teaching Hospital, Anuradhapura, Sri Lanka; 5 Department of Pharmacology, Faculty of Medicine, University of Kelaniya, Ragama, Sri Lanka; 6 Department of Medicine, Faculty of Medicine, University of Kelaniya, Ragama, Sri Lanka

**Keywords:** Thalassaemia, β-Thalassaemia major, Haemoglobin E β-thalassaemia, Transfusion, Quality of life

## Abstract

**Background:**

Thalassaemia is a chronic disease without an effective cure in a majority. The clinical management has improved considerably during recent years; however, minimal attempts are made to up lift the quality of life among patients, especially in developing countries. Here we aim to describe and compare and to determine factors associated with health related quality of life among patients with transfusion dependent β-thalassaemia major and haemoglobin E β-thalassemia in Sri Lanka.

**Methods:**

A case control study was conducted in the three largest thalassaemia centres of Sri Lanka. All patients with transfusion dependent β-thalassaemia (β-thalassaemia major and haemoglobin E β-thalassaemia) aged 5–18 years were recruited as cases whilst a randomly selected group of children without chronic diseases were recruited as controls. Socio-demographic and clinical data were collected using an interviewer-administered questionnaire and health related quality of life was measured using the validated Paediatric Quality of Life Inventory Version 4.0.

**Results:**

Two hundred and seventy one patients with transfusion dependent β-thalassaemia (male-49.1%; mean age- 10.9 ± 3.6 years) and 254 controls (male-47.2%; mean age- 10.4 ± 3.5 years) were recruited. Mean health-related quality of life scores were significantly lower in patients compared to controls (72.9 vs. 91.5, *p* < 0.001). Of the patients, 224 (84%) had β-thalassaemia major and 43 (16%) had haemoglobin E β-thalassaemia. Quality of life scores in psychological health (*p* < 0.05), emotional functioning (*p* < 0.05) and social functioning (*p* < 0.05) were significantly lower in patients with haemoglobin E β-thalassaemia compared to β-thalassaemia major. Splenectomy (*p* < 0.05), short stature (*p* < 0.05), under nutrition (*p* < 0.05) and longer hospital stays (*p* < 0.05) were significantly associated with lower quality of life scores.

**Conclusions:**

Despite improvements in management, the quality of life among patients with β-thalassaemia still remains low. This is more pronounced in the subset of patients with haemoglobin E β-thalassaemia. Splenectomy, short stature, undernutrition and longer hospital stays were significantly associated with poor quality of life. It is timely, even in developing countries, to direct emphasis and to take appropriate steps to improve standards of living and quality of life of patients with β-thalassaemia.

## Introduction

β-Thalassaemia is one of the most common monogenic diseases in the world [1, 2]. Patients with severe forms present during infancy with worsening anaemia and are transfusion dependent for life [3]. Allogenic stem cell transplantation remains the only cure however, is not suitable to a majority of patients due to limitations in matched donors and cost [4]. Numerous attempts in pre-clinical and early clinical studies that aim to discover a cure for all patients have met with variable success thus far [5–8]. Therefore, a majority of patients with transfusion dependent β-thalassaemia (TDBT) are managed medically with regular blood transfusions and iron chelation therapy for life [9].

Medical management of TDBT has improved substantially during the past decade due to availability of safe blood products and effective oral iron chelators [10]. Uniform guidelines which are published and frequently updated by the Thalassaemia International Federation (TIF) are available to guide treatment of these patients [11]. These guidelines advocate 2–5 weekly blood transfusions to maintain a pre-transfusion haemoglobin level between 9.0–10.5 g/dl and regular iron chelation therapy to achieve a serum ferritin value below 1000 ng/dL. Through these management strategies, patients with TDBT live longer - commonly into the fifth decade - and have lesser rates of medical complications [12].

The global distribution of thalassaemia is uneven and show a higher prevalence in tropical regions, that include Mediterranean, middle-east and south and south-east Asia [13]. This is believed to be due to the selective advantage among carriers of β-thalassaemia against *Plasmodium falciparum* malaria which was highly prevalent in these regions [14]. Sri Lanka is a South Asian country located within the tropical thalassaemia belt [15]. The gene frequency of β-thalassaemia in Sri Lanka is 2.8% and approximately 2000 patients with TDBT are being treated in thalassaemia centres across the country [16]. However, a vast majority (over two-thirds of patients) are managed in three large tertiary referral centres, namely Kurunegala, Anuradhapura and Ragama thalassaemia centres [17]. All three centres have unrestricted access to blood products through a state-run National Blood Transfusion service, provide iron chelation medication to all patients free of charge and follow the TIF guidelines, hence, can be considered as units providing optimal care for patients with TDBT [18].

Despite improvement in medical care, one aspect that has been neglected in Sri Lanka and all over the world is to take necessary steps to improve the standards of living and quality of life of patients with thalassaemia [19]. Several studies done in the past when the medical care of thalassaemia was relatively poor show that patients with TDBT experienced significantly poor quality of life [20–23]. However, large scale studies assessing the same since improvements in medical care specially confined to paediatric population are sparse. Therefore, in this study we aim to describe and compare and to determine factors associated with health related quality of life (HRQoL) among patients with transfusion dependent β-thalassaemia major and haemoglobin E β-thalassemia in Sri Lanka and to compare it with non-thalassaemia controls.

## Methods

This case control study was conducted in the three largest thalassaemia centres of Sri Lanka from September 2017 to March 2018. All patients with TDBT aged between 5 to 18 years attending Kurunegala, Anuradhapura and Ragama thalassaemia centres during the study period were recruited as cases. The diagnosis of thalassaemia was based on haemoglobin sub-type quantification using high performance liquid chromatography and ‘transfusion dependency’ was defined as receiving transfusions more frequently than 6-weekly. This group represents more than half of the children with TDBT aged between 5 to 18 year in Sri Lanka. A group of children without chronic diseases who were admitted to the same hospitals for acute non-life-threatening illnesses were recruited as controls. Controls were selected by simple random sampling using a random number table until same number of controls as cases were recruited. Parents of participants were briefed about the study and informed written consent from guardians and assent from children over 12 years were taken before recruiting into the study.

Data were collected using three study instruments. Firstly, an interviewer-administered questionnaire was completed by a trained data collector by interviewing patients and their parents to gather data on basic demographics, parental education level and occupations, monthly family income, previous hospital stays, transfusion history and transfusion reactions. Secondly, a data collection form was completed from cases by perusal of clinical records and physical examinations by trained doctors to gather information on pre-transfusion haemoglobin levels, volume of blood transfusions, anthropometric measurements, liver and spleen sizes and disease complications.

Next, all participants were given the self-administered Paediatric Quality of Life Inventory Version 4.0 (PedsQL 4.0) Generic core scales to assess the HRQoL. We used the previously translated and validated Sinhalese (native language of Sri Lanka) version of this questionnaire [24–26]. Three separate questionnaires for different age groups were used. Questionnaires for age groups 5–7 years and 8–12 years were answered by parents whereas questionnaires for age group 13–18 were answered by children themselves. Each questionnaire contained questions to assess HRQoL in four dimensions – physical functioning, emotional functioning, social functioning and school functioning. The psychosocial health summary score was calculated by averaging scores for emotional, social, and school functioning dimensions and the overall HRQoL score was calculated by averaging scores for all four dimensions. In the event of incomplete questionnaires, mean of the completed items were imputed if 50% or more items were completed. If more than 50% of the items in the scale were missing, the scale scores were not be computed. Scores in each category ranged from 0 to 100 with higher scores representing better HRQoL.

Data were analysed using IBM SPSS statistics 25.0 for windows. Categorical data were expressed as frequencies (percentages) and continuous data on HRQoL were expressed as median (interquartile range) and mean (standard deviation). Independent sample Student’s t-test was used to compare means and Mann-Whitney U test was used to compare medians. χ^2^ test was used to test for significance in categorical variables. Pearson’s correlation coefficient and Spearman’s rank correlation were used to assess correlations between quantitative variables and HRQoL scores. When analysing for associations between categorical and dichotomised variables and the quality of life, mean HRQoL scores were initially compared using Student’s t-test. Then the adjusted *p*-values for significant associations were calculated using multiple linear regressions after adjusting for age, sex and type of thalassaemia. Statistical significance was defined as *p* < 0.05. Ethical approval was obtained from the Ethics Review Committee of University of Kelaniya, Sri Lanka.

## Results

**Fig. 1:**
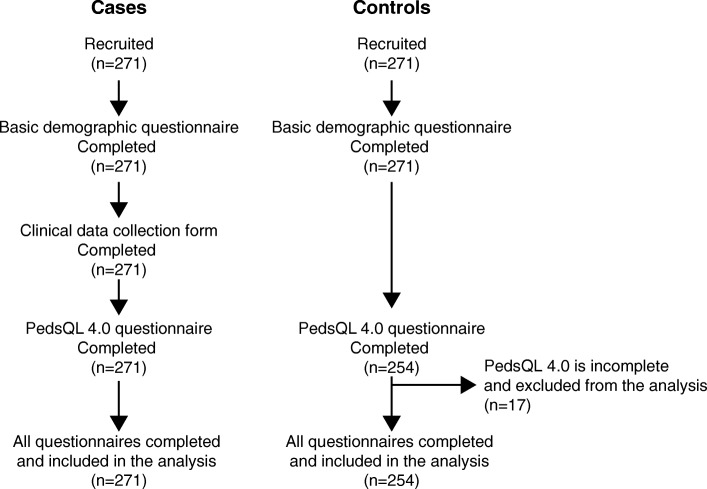
Flow diagram demonstrating recruitment of cases and controls

A total of 271 patients with TDBT and an equal number of non-thalassaemia controls were recruited into the study. All patients and 254 (93.7%) controls completed questionnaires and were included in the analysis (Fig. 1). Age and sex distributions of patients and controls were similar. Mean ages were 10.9 ± 3.6 and 10.4 ± 3.5 years respectively for patients and controls (*p* = 0.08). 133 (49.1%) patients and 120 (47.2%) controls were males (χ^2^ = 0.17, *p* = 0.67). Of the patients, a majority (84%) had homozygous β-thalassaemia major where as 16% had haemoglobin (Hb) E β-thalassaemia. Clinical characteristics of the patient population are shown in Table 1.

**Table 1:** Clinical characteristics of patients with transfusion dependent β-thalassaemia

Characteristic	*N* = 271
	Mean (±SD)
Duration of the disease (years)	9.8 (±3.8)
Average pretransfusion haemoglobin (g/dL)	8.4 (±1.1)
Annual transfusion requirement (ml/kg/year)	235 (±76)
Average serum ferritin (ng/mL)	1993 (±1844)
Duration of hospital stay (days)	2.29 (±1.0)
Distance from home to hospital (km)	54.8 (±48)
	Frequency (%)
Age groups	
5–7 years	68 (25.1%)
8–12 years	116 (42.8%)
13–18 years	87 (32.1%)
Sub-type of thalassaemia	
β-thalassaemia major	228 (84.1%)
HbE β-thalassaemia	43 (15.9%)
Thalassaemia treatment centre	
Kurunegala	155 (57.2%)
Anuradhapura	79 (29.1%)
Ragama	37 (13.7%)
Frequency of blood transfusions	
> 4 weekly	29 (10.7%)
4 weekly	208 (76.8%)
3 weekly	33 (12.2%)
< 3 weekly	1 (0.4%)
Average pretransfusion haemoglobin	
< 7.0 g/dl	32 (11.8%)
7.0–8.9 g/dl	132 (48.7%)
9.0–10.5 g/dl	99 (36.5%)
> 10.5 g/dl	8 (3.0%)
Annual transfusion requirement^a^	
< 200 ml/kg/year	109 (42.6%)
201–250 ml/kg/year	41 (16.0%)
251–300 ml/kg/year	55 (21.5%)
> 300 ml/kg/year	51 (19.9%)
Spleen status	
No splenomegaly	179 (66.1%)
Splenomegaly of 1–3 cm	74 (27.3%)
Splenomegaly of > = 4 cm	11 (4.1%)
Splenectomised	7 (2.5%)
Liver status	
No hepatomegaly	188 (69.3%)
Hepatomegaly of 1–2 cm	67 (24.7%)
Hepatomegaly > = 3 cm	16 (5.9%)
Serum Ferritin^b^	
< 1000 ng/mL	83 (31.6%)
1001–2500 ng/mL	122 (46.4%)
2501–5000 ng/mL	38 (14.4%)
> 5000 ng/mL	20 (7.6%)
Iron chelator medication	
No chelation	1 (0.4%)
Deferasirox	163 (60.1%)
Deferoxamine	29 (10.7%)
Deferiprone	3 (1.1%)
Deferasirox + Deferoxamine	75 (27.7%)
Complications	
Thalassaemia facies	92 (33.9%)
Skin pigmentation	57 (21.0%)
Short stature^c^	117 (48.0%)
Undernutrition^d^	85 (35.1%)
Type 1 diabetes	5 (1.8%)
Hypothyroidism	11 (4.1%)
Cardiomyopathy	2 (0.7%)
Elevated transaminases	58 (21.4%)
Cirrhosis	0
Allergic reaction to transfusion	60 (22.1%)
Hepatitis C infection	60 (22.1%)
Abdominal scars	66 (24.4%)
Hearing impairment	4 (1.5%)
Visual impairment	19 (7.0%)

Data missing from: ^a^15 patients; ^b^8 patients; ^c^27 patients; and ^d^29 patients

### Health-related quality of life among patients with transfusion dependent β-thalassaemia and controls

**Fig. 2:**
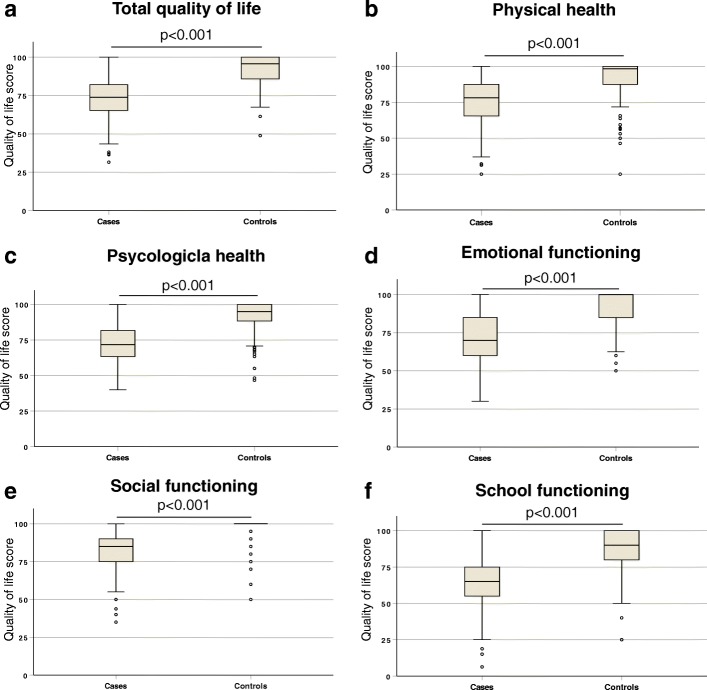
Health-related quality of life scores in different dimensions among patients with transfusion dependent β-thalassaemia and controls; (**a**) Total quality of life, (**b**) Physical health, (**c**) Psychological health, (**d**) Emotional functioning, (**e**) Social functioning and (**f**) School functioning. Each box plot shows interquartile range, middle horizontal bars demonstrate respective median and error bars show range; outliers are marked in circles. *P*-values are calculated using Mann-Whitney U test

Comparison of HRQoL between patients and controls revealed significant differences (Fig. 2). Patients with TDBT had significantly lower mean overall HRQoL scores compared to controls (72.9 vs. 91.5, *p* < 0.001). Similarly, mean quality of life scores in individual dimensions – physical health (74.6 vs. 91.4, *p* < 0.001), psychological health (72.0 vs. 91.4, *p* < 0.001), emotional functioning (70.5 vs. 90.7, *p* < 0.001), social functioning (80.8 vs. 96.8, *p* < 0.001) and school functioning (65.1 vs. 87.2, *p* < 0.001) were significantly lower in patients with TDBT compared to controls. The mean overall HRQoL scores in three centres were different with highest in the Ragama centre (79.1 ± 13.9) followed by Anuradhapura (72.6 ± 11.3) and Kurunegala (71.5 ± 12.3) centres (*p* < 0.01).

### Health-related quality of life among patients with β-thalassaemia major and haemoglobin E β-thalassaemia

**Fig. 3:**
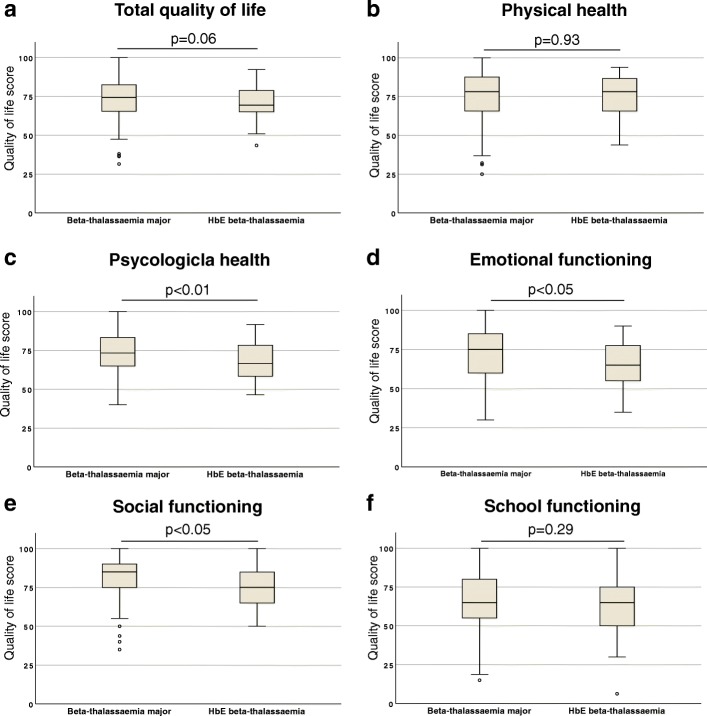
Health-related quality of life scores in different dimensions among patients with β-thalassaemia major and HbE β-thalassaemia; (**a**) Total quality of life, (**b**) Physical health, (**c**) Psychological health, (**d**) Emotional functioning, (**e**) Social functioning and (**f**) School functioning. Each box plot shows interquartile range, middle horizontal bars demonstrate respective median and error bars show range; outliers are marked in circles. *P*-values are calculated using Mann-Whitney U test

Next, we evaluated the HRQoL scores among patients with β-thalassaemia major and HbE β-thalassaemia (Fig. 3). Overall mean HRQoL scores were lower in patients with HbE β-thalassaemia (70.1 ± 11.2) compared to β-thalassaemia major (73.4 ± 12.6), however the difference was not statistically significant (*p* = 0.11). Contrarily, quality of life scores in psychological health (67.9 vs. 72.8, *p* < 0.05), emotional functioning (64.7 vs. 71.6, *p* < 0.05) and social functioning (76.5 vs. 81.6, *p* < 0.05) were significantly lower in patients with HbE β-thalassaemia compared to β-thalassaemia major.

### Correlation between socio-demographic and clinical characteristics and health-related quality of life

**Fig. 4:**
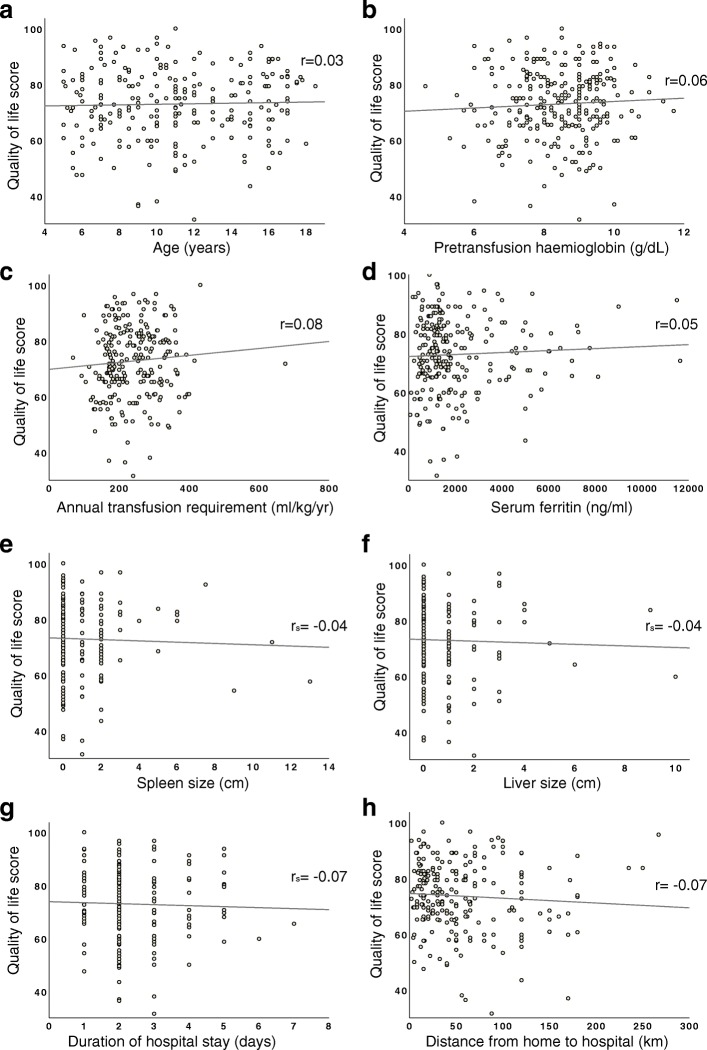
Correlation between overall health-related quality of life score and socio-demographic and clinical parameters among patients with transfusion dependent β-thalassaemia; (**a**) Age, (**b**) Pretransfusion haemoglobin, (**c**) Annual transfusion requirement, (**d**) Serum ferrtin, (**e**) Spleen size, (**f**) Liver size, (**g**) Duration of hospital stay and (**h**) Distance from home to hospital. Each dot in scatter plots represents individual patients and correlation were quantified using Pearson correlation coefficient (r, for continuous data) or Spearman correlation coefficient (r_s,_ for discrete or ordinal data)

Then, we analysed the correlation between HRQoL and a number of socio-demographic and clinical characteristics among patients with TDBT (Fig. 4) Age (*r* = 0.03), pretransfusion haemoglobin levels (*r* = 0.06), annual transfusion requirements (*r* = 0.08) and serum ferritin levels (*r* = 0.05) showed weak positive correlations with overall quality of life scores. Conversely, size of the liver (r_s_ = 0.04) and spleen (r_s_ = 0.04), duration of hospital stay (r_s_ = 0.07) and distance from home to hospital (*r* = 0.07) had weak negative correlations with overall HRQoL scores. Age (*r* = 0.13, *p* < 0.05), duration of the disease (*r* = 0.17, *p* < 0.01) and annual transfusion requirements (*r* = 0.16, *p* < 0.05) demonstrated significant positive correlations with the emotional functioning score whilst the distance from home to hospital had significant negative correlation with school functioning score (*r* = − 0.14, *p* < 0.05) (Table 2).

**Table 2:** Correlation between socio-demographic and clinical characteristics with health-related quality of life

	Total quality of life score	Physical functioning score	Psychosocial health summary score	Emotional functioning score	Social functioning score	School functioning score
Age^$^ (*n* = 271)	0.029	0.082	(−) 0.004	0.126*	(−) 0.046	(−) 0.084
Duration of disease^$^ (*n* = 271)	0.060	0.075	0.053	0.171**	0.034	(−) 0.088
Average pretransfusion haemoglobin^$^ (*n* = 271)	0.056	(−) 0.014	0.086	0.083	0.072	0.032
Annual transfusion requirement^$^ (*n* = 256)	0.075	0.024	0.095	0.157*	0.041	0.016
Average serum ferritin^$^ (*n* = 263)	0.050	0.105	0.008	0.044	0.048	(−) 0.071
Spleen size^#^ (*n* = 271)	(−) 0.038	0.008	(−) 0.054	(−) 0.057	(−) 0.021	(−) 0.059
Liver size^#^ (*n* = 271)	(−) 0.044	(−) 0.005	(−) 0.062	(−) 0.025	(−) 0.034	(−) 0.082
Duration of hospital stay^#^ (*n* = 242)	(−) 0.064	(−) 0.009	(−) 0.073	(−) 0.018	(−) 0.097	(−) 0.106
Distance from home to hospital^$^ (*n* = 244)	(−) 0.068	(−) 0.002	(−) 0.079	(−) 0.007	(−) 0.053	(−) 0.143*

Respective correlation coefficients are shown in the table. ^$^Pearson correlation coefficient; ^#^Spearman correlation coefficient. *p < 0.05; ** *p* < 0.01

### Clinical determinants of health-related quality of life

Next, we evaluated the significant association between a number of dichotomized clinical characteristics and quality of life (Table 3). Patients who underwent splenectomy had lower quality of life scores compared to the ones who did not undergo the procedure. These differences were statistically significant after adjusting for age, sex and type of thalassaemia with regards to overall HRQoL scores (*p* < 0.05) and in several dimensions; psychological health (*p* < 0.05), emotional functioning (*p* < 0.01) and social functioning (*p* < 0.05). Both short stature (*p* < 0.01) and undernutrition (*p* < 0.05) were significantly associated with lower overall quality of life scores. Additionally, children with short stature had significantly lower scores in psychological health (*p* < 0.01), social functioning (*p* < 0.01) and school functioning (*p* < 0.01) compared to the patients who had normal heights. Similarly, undernutrition was associated with lower scores in psychological health (*p* < 0.05) and school functioning (*p* < 0.01). In this study we also observed that presence of thalassaemic facies was associated with higher overall HRQoL (*p* < 0.001) and higher scores in physical health (*p* < 0.01), psychological health (*p* < 0.01), emotional functioning (*p* < 0.01) and school functioning (*p* < 0.01) dimensions.

**Table 3:** Associations between clinical characteristics and health-related quality of life among patients with transfusion dependent β-thalassaemia

	Total quality of life score (Mean ± SD)	Physical functioning score (Mean ± SD)	Psychosocial health summary score (Mean ± SD)	Emotional functioning score (Mean ± SD)	Social functioning score (Mean ± SD)	School functioning score (Mean ± SD)
Average pretransfusion haemoglobin						
> 9.0 g/dl (*n* = 107)	73.9 (±12.4)	74.6 (±15.6)	73.6 (±12.4)	71.8 (±15.3)	82.6 (±15.9)	66.3 (±17.6)
< 9.0 g/dl (*n* = 164)	72.2 (±12.5)	74.5 (±15.4)	71.0 (±12.7)	69.7 (±17.0)	79.6 (±14.6)	64.3 (±17.1)
*p-value*	*0.26*	*0.97*	*0.10*	*0.31*	*0.10*	*0.36*
Annual transfusion requirement						
< 250 ml/kg/year (*n* = 150)	71.8 (±13.0)	73.9 (±16.3)	70.8 (±12.7)	68.9 (±16.4)	80.5 (±15.6)	63.4 (±17.7)
> 250 ml/kg/year (*n* = 106)	73.8 (±12.1)	74.8 (±15.1)	73.3 (±12.6)	72.3 (±16.6)	81.1 (±15.2)	66.4 (±16.3)
*p-value*	*0.20*	*0.64*	*0.13*	*0.10*	*0.75*	*0.16*
Liver status						
No hepatomegaly (*n* = 188)	73.5 (±11.9)	74.8 (±15.0)	72.8 (±12.2)	71.0 (±16.1)	81.4 (±14.4)	66.3 (±17.4)
Hepatomegaly (*n* = 83)	71.5 (±13.6)	74.0 (±16.6)	70.4 (±13.4)	69.4 (±17.0)	79.3 (±16.7)	62.3 (±16.9)
*p-value*	*0.22*	*0.70*	*0.15*	*0.47*	*0.28*	*0.08*
Spleen status						
No splenomegaly (*n* = 179)	73.7 (±12.1)	74.8 (±15.5)	73.0 (±13.3)	71.8 (±16.1)	81.6 (±14.1)	66.2 (±17.6)
Splenomegaly (*n* = 85)	72.0 (±13.1)	74.8 (±14.9)	70.8 (±13.3)	69.1 (±16.4)	80.0 (±16.6)	63.2 (±16.7)
*p-value*	*0.30*	*0.98*	*0.17*	*0.21*	*0.41*	*0.18*
Splenectomy status						
No splenomegaly (*n* = 179)	73.7 (±12.1)	74.8 (±15.5)	73.0 (±13.3)	71.8 (±16.1)	81.6 (±14.1)	66.2 (±17.6)
Splenectomised (*n* = 7)	62.4 (±8.1)	64.4 (±20.5)	61.7 (±6.7)	55.7 (±14.5)	70.1 (±20.7)	59.2 (±15.3)
*p-value*	*< 0.05*	*0.08*	*< 0.05*	*< 0.05*	*< 0.05*	*0.30*
B	(−)12.0		(−)12.0	(−)18.0	(−)10.5	
*p-value (adjusted)*	*< 0.05*		*< 0.05*	*< 0.01*	*< 0.05*	
Serum ferritin						
< 1000 ng/mL (*n* = 83)	72.0 (±12.8)	73.6 (±15.3)	71.1 (±12.8)	69.9 (±16.6)	79.4 (±14.8)	64.3 (±18.1)
> 1000 ng/mL (*n* = 180)	73.1 (±12.2)	74.8 (±15.7)	72.3 (±12.4)	70.7 (±16.4)	81.4 (±15.1)	65.0 (±16.9)
*p-value*	*0.49*	*0.57*	*0.49*	*0.72*	*0.30*	*0.78*
Skin pigmentation						
No (*n* = 214)	73.0 (±12.5)	74.5 (±15.5)	72.4 (±12.5)	71.2 (±16.2)	80.7 (±15.0)	65.5 (±16.5)
Yes (*n* = 57)	72.4 (±12.4)	74.7 (±15.5)	70.6 (±12.8)	68.2 (±17.0)	81.1 (±15.8)	63.7 (±20.2)
*p-value*	*0.75*	*0.91*	*0.32*	*0.22*	*0.85*	*0.49*
Diabetes						
No (*n* = 266)	73.0 (±12.4)	74.7 (±15.5)	72.2 (±12.6)	70.6 (±16.4)	80.9 (±15.1)	65.2 (±17.3)
Yes (*n* = 5)	66.5 (±14.7)	68.1 (±16.5)	66.3 (±15.1)	68.0 (±17.5)	73.0 (±19.2)	58.0 (±19.5)
*p-value*	*0.24*	*0.34*	*0.30*	*0.72*	*0.24*	*0.35*
Hypothyroidism						
No (*n* = 260)	73.0 (±12.5)	74.9 (±15.4)	72.1 (±12.7)	70.7 (±16.4)	80.7 (±15.3)	65.2 (±17.0)
Yes (*n* = 11)	69.3 (±12.2)	67.2 (±16.7)	70.5 (10.0)	67.2 (±16.7)	82.2 (±11.4)	61.4 (±23.9)
*p-value*	*0.33*	*0.11*	*0.68*	*0.49*	*0.74*	*0.47*
Short stature						
No (*n* = 127)	74.8 (±13.1)	75.9 (±16.8)	74.4 (±13.0	71.2 (±17.0)	83.9 (±14.5)	68.0 (±17.5)
Yes (*n* = 117)	71.0 (±12.1)	72.9 (±14.6)	69.9 (±12.1)	70.1 (±16.0)	77.9 (±15.6)	61.8 (±16.7)
*p-value*	*< 0.05*	*0.14*	*< 0.01*	*0.58*	*< 0.01*	*< 0.01*
B	(−)4.7		(−)5.3		(−)6.4	(−)6.3
*p*-value (adjusted)	< 0.01		< 0.01		< 0.01	< 0.01
Undernutrition						
No (*n* = 157)	74.3 (±12.7)	75.9 (±16.3)	73.7 (±12.4)	71.4 (±15.9)	82.1 (±14.7)	67.4 (±17.3)
Yes (*n* = 85)	70.7 (±12.4)	72.1 (±14.6)	69.9 (±13.1)	69.7 (±17.4)	79.3 (±16.2)	60.6 (±16.9)
*p-value*	*< 0.05*	*0.07*	*< 0.05*	*0.42*	*0.17*	*< 0.01*
B	(−)3.7		(−)3.7			(−)6.5
*p-value (adjusted)*	*< 0.05*		*< 0.05*			*< 0.01*
Hepatitis C infection						
No (*n* = 211)	71.7 (±12.0)	73.6 (±15.0)	70.7 (±12.3)	69.3 (±16.0)	79.1 (±15.2)	63.8 (±17.4)
Yes (*n* = 60)	77.2 (±13.1)	77.9 (±16.8)	76.9 (±12.4)	74.7 (±17.2)	86.5 (±13.8)	69.6 (±16.3)
*p-value*	*< 0.01*	*0.05*	*< 0.01*	*< 0.05*	*< 0.01*	*< 0.05*
B	5.5		6.3	5.1	7.5	6.3
*p-value (adjusted)*	*< 0.01*		*< 0.01*	*< 0.05*	*< 0.01*	*< 0.05*
Abdominal scars						
No (*n* = 205)	72.8 (±13.0)	74.5 (±15.8)	71.8 (±13.1)	70.1 (±16.9)	80.3 (±15.8)	65.4 (±17.3)
Yes (*n* = 66)	73.2 (±10.7)	74.8 (±14.7)	72.7 (±11.0)	71.8 (±14.6)	82.1 (±13.0)	64.1 (±17.5)
*p-value*	*0.78*	*0.86*	*0.64*	*0.47*	*0.41*	*0.59*
Thalassaemia facies						
No (n = 179)	71.0 (±12.5)	72.5 (±16.2)	70.3 (±12.4)	68.5 (±16.4)	79.7 (±15.8)	63.1 (±16.5)
Yes (*n* = 92)	76.5 (±11.6)	78.5(±13.2)	75.4 (±12.3)	74.4 (±15.6)	82.8 (±13.6)	68.9 (±18.2)
*p-value*	*< 0.01*	*< 0.01*	*< 0.01*	*< 0.01*	*0.10*	*< 0.01*
B	5.6	5.7	5.5	5.8		6.6
*p-value (adjusted)*	*< 0.001*	*< 0.01*	*< 0.01*	*< 0.01*		*< 0.01*
Hearing problems						
Yes (*n* = 4)	73.9 (±12.7)	75.0 (±8.8)	73.3 (±15.3)	80.0 (±10.8)	83.7 (±17.0)	56.2 (±18.8)
No (*n* = 267)	72.9 (±12.5)	74.5 (±15.6)	72.0 (±12.6)	70.4 (±16.4)	80.7 (±15.2)	65.2 (±17.3)
*p-value*	*0.87*	*0.95*	*0.84*	*0.24*	*0.69*	*0.30*
Visual problems						
No (*n* = 252)	73.0 (±12.5)	74.4 (±15.7)	72.2 (±12.6)	70.7 (±16.5)	80.7 (±15.5)	65.5 (±16.5)
Yes (*n* = 19)	72.0 (±12.0)	76.0 (±11.8)	70.0 (±12.8)	68.4 (±15.0)	81.3 (±9.9)	60.0 (±25.4)
*p-value*	*0.75*	*0.68*	*0.47*	*0.55*	*0.88*	*0.18*
Elevated transaminases						
No (*n* = 213)	73.2 (±12.6)	74.4 (±15.8)	72.6 (±12.7)	70.2 (±16.4)	81.5 (±15.1)	66.3 (±17.1)
Yes (*n* = 58)	71.9 (±11.8)	75.1 (±14.2)	70.2 (±12.2)	71.7 (±16.2)	78.1 (±15.2)	60.7 (±17.7)
*p-value*	*0.48*	*0.75*	*0.20*	*0.54*	*0.12*	*< 0.05*
B						(−)5.2
*p-value (adjusted)*						*< 0.05*
Iron chelator medication						
Desferioxamine (*n* = 29)	74.2 (±12.2)	79.3 (±13.9)	71.7 (±13.7)	71.4 (±15.5)	79.1 (±15.9)	64.7 (±17.5)
Deferasirox (*n* = 163)	71.9 (±12.8)	72.9 (±16.4)	71.4 (±12.5)	69.7 (±17.1)	79.7 (±15.3)	65.0 (±17.4)
*p-value*	*0.37*	*0.05*	*0.88*	*0.61*	*0.84*	*0.92*

*p*-values were calculated using Student’s t-test

B - unstandardized coefficient of regression

*p*-value (adjusted) were calculated using multiple linear regression after adjusting for age, sex and type of thalassaemia

### Socio-demographic determinants of health-related quality of life

Finally, we examined the associations between dichotomized socio-demographic characteristics and the HRQoL (Table 4). Higher level of education in the father was significantly associated with higher HRQoL scores (*p* < 0.01). This trend was observed in all dimensions of physical health (*p* < 0.01) and psychological health (*p* < 0.001). Similarly, patients whose fathers were engaged in skilled or professional occupations had significantly higher overall (*p* < 0.05), psychological health (*p* < 0.05), social functioning (*p* < 0.05) and school functioning (*p* < 0.05) HRQoL scores compared to children of fathers with unskilled occupations. Furthermore, significantly lower school functioning HRQoL scores were reported by patients who spend longer than 2 days (*p* < 0.01) and live over 100 km away (*p* < 0.001) from the hospital.

**Table 4:** Associations between socio-demographic characteristics and health-related quality of life among patients with transfusion dependent β-thalassaemia

	Total quality of life score (Mean ± SD)	Physical functioning score (Mean ± SD)	Psychosocial health summary score (Mean ± SD)	Emotional functioning score (Mean ± SD)	Social functioning score (Mean ± SD)	School functioning score (Mean ± SD)
Education level of the mother						
Below O/L (*n* = 184)	72.8 (±12.9)	74.8 (±15.8)	71.9 (±12.9)	70.7 (±17.0)	80.4 (±15.6)	64.9 (±17.9)
A/L or higher (*n* = 87)	73.0 (±11.4)	74.1 (±14.9)	72.4 (±12.0)	70.2 (±15.1)	81.6 (±14.3)	65.4 (±16.0)
*p-value*	*0.88*	*0.73*	*0.75*	*0.84*	*0.55*	*0.84*
Education level of the father						
Below O/L (*n* = 199)	71.5 (±13.2)	73.1 (±16.1)	70.6 (±13.3)	69.4 (±17.3)	79.5 (±16.0)	63.4(±18.3)
A/L or higher (*n* = 72)	76.8 (±9.3)	78.6 (±12.7)	75.9 (±9.4)	73.6 (±13.0)	84.3 (±11.9)	69.8 (±13.4)
*p-value*	*< 0.001*	*< 0.01*	*< 0.001*	*< 0.05*	*< 0.01*	*< 0.01*
B	5.1	5.5	5.1	3.9	4.7	6.2
*p-value (adjusted)*	*< 0.01*	*< 0.01*	*< 0.01*	*< 0.05*	*< 0.05*	*< 0.01*
Mother’s occupation						
Housewife/ Unemployed (*n* = 228)	72.9 (±12.5)	74.7 (±15.1)	72.0 (±12.8)	70.4 (±16.7)	81.0 (±15.6)	65.0 (±17.5)
Employed (*n* = 43)	72.6 (±12.5)	73.8 (±17.6)	72.4 (±11.6)	71.1 (±14.7)	79.8 (±12.9)	65.6 (±16.6)
*p-value*	*0.85*	*0.72*	*0.84*	*0.78*	*0.64*	*0.81*
Father’s occupation						
Unemployed/ Unskilled (*n* = 187)	71.7 (±12.3)	73.6 (±15.6)	70.8 (±12.5)	70.0 (±16.6)	79.3 (±15.8)	63.2 (±16.8)
Skilled/ Professional (*n* = 84)	75.4 (±12.4)	76.6 (±15.1)	74.8 (±12.4)	71.6 (±15.9)	83.9 (±13.2)	69.2 (±17.7)
*p-value*	*< 0.05*	*0.14*	*< 0.05*	*0.47*	*< 0.05*	*< 0.05*
B	3.7		4.0		4.4	5.7
*p-value (adjusted)*	*< 0.05*		*< 0.05*		*< 0.05*	*< 0.05*
Monthly family income (LKR)						
< 50,000 (*n* = 253)	72.6 (±12.4)	74.2 (±15.4)	71.7 (±12.6)	70.4 (±16.6)	80.4 (±15.2)	64.5 (±17.3)
> 50,000 (*n* = 17)	77.6 (±12.6)	79.9 (±16.1)	76.1 (±12.3)	71.7 (±13.4)	85.0 (±14.6)	72.6 (±15.8)
*p-value*	*0.10*	*0.14*	*0.16*	*0.75*	*0.23*	*0.06*
Average duration of hospital stay						
1 day (*n* = 41)	75.9 (±12.2)	77.2 (±15.1)	75.1 (±12.5)	71.3 (±18.7)	83.2 (±15.9)	71.0 (±12.3)
> 1 day (*n* = 230)	72.3 (±12.4)	74.1 (±15.5)	71.5 (±12.6)	70.4 (±15.9)	80.3 (15.0)	64.0 (±17.9)
*p-value*	*< 0.05*	*0.23*	*0.09*	*0.73*	*0.27*	*< 0.01*
B	(−)4.2					(−)8.5
*p-value (Adjusted)*	*0.05*					*< 0.01*
Distance from home to hospital						
< 100 km (*n* = 206)	74.1 (±12.3)	75.7 (±15.3)	73.3 (±12.4)	71.4 (±16.1)	81.6 (±15.2)	67.2 (±15.7)
> 100 km (*n* = 38)	70.8 (±12.7)	74.14.7)	69.1 (±13.1)	71.0 (±16.4)	80.1 (±14.5)	55.4 (±22.1)
*p-value*	*0.13*	*0.78*	*0.06*	*0.88*	*0.59*	*< 0.01*
B						(−)11.0
*p-value (adjusted)*						*< 0.001*

*p*-values were calculated using Student’s t-test

B - unstandardized coefficient of regression

*p*-value (adjusted) were calculated using multiple linear regression after adjusting for age, sex and type of thalassaemia

## Discussion

The outlook of thalassaemia has remarkably transformed over the past few years from a life-threatening fatal disease to a chronic disease with disability [12]. The clinical management has improved extensively during recent years even in developing countries however, minimal attempts are made to up lift the quality of life among patients [27]. Hence it is important to assess the HRQoL in patients with β-thalassaemia and to identify factors that lead to poor quality of life. In this study we evaluated the HRQoL among a large cohort of paediatric patients with transfusion dependent β-thalassaemia major and HbE β-thalassaemia in comparison to non-thalassaemia controls. Furthermore, we examined the individual factors that might influence the HRQoL in patients with TDBT in detail.

In this study we assessed the HRQoL using the Paediatric Quality of Life Inventory Version 4.0 (PedsQL 4.0) Generic core scales. We used this questionnaire instead of the recently validated disease specific quality of life questionnaire designed for transfusion dependent thalassaemia (Transfusion-dependent QoL questionnaire- TranQol) due to number of reasons [28]. Firstly, a validated Sinhalese translation is available only for PedsQL 4.0 questionnaire. Secondly, we compared the HRQoL scores of patients with β-thalassaemia with that of healthy controls therefore, a generic questionnaire was deemed more suitable. Also, PedsQL 4.0 questionnaire was easy to use and has been widely used among patients with thalassaemia previously [20, 29].

Our results showed that patients with TDBT have significantly lower HRQoL scores in all dimensions when compared to non-thalassaemia controls. This is not surprising considering the chronic nature of the disease, however, is concerning as the HRQoL scores are still significantly lower despite achieving remarkable improvements in medical care. Our results are comparable to the findings of a study done in Thailand a decade ago which reported a mean HRQoL score of 76 ± 11 using the same scale used in our study [20]. This suggests that the improvements in medical care may not have contributed substantially to upgrade the quality of life of these patients. However, the quality of life scores reported in these studies are significantly higher than the scores reported among patients in Egypt [29]. This is probably due to the difference in the clinical management where patients in south and southeast Asia receiving better levels of transfusion and chelation compared to patients in Africa.

Another important observation made in this study is that the patients with HbE β-thalassaemia have a lower HRQoL scores when compared to β-thalassaemia major; significant differences were observed in psychological health, emotional functioning and social functioning dimensions. This is rather surprising given the fact that HbE β-thalassaemia is considered as a minor disease [30]. However, we have previously shown that patients with HbE β-thalassaemia have lower mean pretransfusion haemoglobin levels and higher rates of hepatosplenomegaly despite receiving similar volumes of blood transfusions [17]. Some or all of these factors might have contributed to lower HRQoL scores observed in patients with HbE β-thalassaemia in our study.

As expected and reported before [20, 31, 32], our results show that higher pretransfusion haemoglobin levels and lower hepatic and splenic sizes are associated with better HRQoL scores. Similarly, longer duration of hospital stay for transfusion and increasing distance to travel from home to hospital were both correlated with lower HRQoL scores. Additionally, short stature and undernutrition were associated with lower HRQoL scores. Notably, both these were associated with lower psychological health scores demonstrating their psychological impact.

Another noteworthy finding of our study is the significantly lower HRQoL in splenectomised patients when compared with non-splenectomised patients. These differences were marked in emotional and social functioning and psychological heath dimensions. Our findings are limited by a small number of patients undergoing splenectomy due to up-dated recommendations however, will significantly support the evidence-base against routine splenectomy in patients with thalassaemia [33].

One interesting finding of this study is the impact of characteristic thalassaemic facies on HRQoL. Thalassaemic facies is long believed to be a disfiguring feature in thalassaemia and is considered as an indication to commence transfusion in non-transfusion dependent patients due to its psychosocial adversity [34]. Nonetheless, we found that the patients with thalassaemia facies have higher HRQoL scores in both physical and psychological health dimensions. Although these results are limited by subjective assessment of thalassaemia facies and other confounders, it is likely that facial characteristics does not have a negative impact on psychological, emotional or social functioning of these patients at least during childhood.

Our results revealed that children with hepatitis C virus infection have significantly higher HRQoL scores in many dimensions tested. This observation is difficult to explain however, could be due to confounders. Similarly, we observed a positive correlation between HRQoL scores and serum ferritin. These correlations were very weak and non-significant therefore, would not have clinical significances.

## Conclusion

In conclusion this study shows that despite improved management, patients with TDBT have significantly lower HRQoL compared to non-thalassaemia controls. More importantly, the patients with HbE β-thalassaemia, which is considered a relatively milder disease, reported lower HRQoL compared to patients with more severe β-thalassaemia major. Splenectomy, short stature, undernutrition, longer hospital stays, and lower paternal education and occupation levels were significantly associated with poor HRQoL. It is timely that necessary steps are taken, even in developing countries, to direct emphasis towards improving standards of living and quality of life of patients with TDBT.

## Data Availability

The datasets used and/or analysed during the current study are available from the corresponding author on reasonable request.

## Abbreviations

HbE: Haemoglobin E
HRQoL: Health related quality of life
PedsQL: Paediatric Quality of Life Inventory
TDBT: Transfusion dependent β-thalassaemia
TIF: Thalassaemia international federation
